# Ten genes and two topologies: an exploration of higher relationships in skipper butterflies (Hesperiidae)

**DOI:** 10.7717/peerj.2653

**Published:** 2016-12-06

**Authors:** Ranjit Kumar Sahoo, Andrew D. Warren, Niklas Wahlberg, Andrew V. Z. Brower, Vladimir A. Lukhtanov, Ullasa Kodandaramaiah

**Affiliations:** 1 School of Biology, Indian Institute of Science Education and Research Thiruvananthapuram, Thiruvananthapuram, Kerala, India; 2 McGuire Center for Lepidoptera and Biodiversity, Florida Museum of Natural History, University of Florida, UF Cultural Plaza, Gainesville, FL, USA; 3 Department of Biology, Lund University, Lund, Sweden; 4 Department of Biology, University of Turku, Turku, Finland; 5 Evolution and Ecology Group, Department of Biology, Middle Tennessee State University, Murfreesboro, TN, USA; 6 Department of Insect Systematics, Zoological Institute of Russian Academy of Sciences, St. Petersburg, Russia; 7 Department of Entomology, St. Petersburg State University, St. Petersburg, Russia

**Keywords:** Skipper butterflies, Hesperiidae, Phylogeny, Contrasting topologies, Incongruence

## Abstract

Despite multiple attempts to infer the higher-level phylogenetic relationships of skipper butterflies (Family Hesperiidae), uncertainties in the deep clade relationships persist. The most recent phylogenetic analysis included fewer than 30% of known genera and data from three gene markers. Here we reconstruct the higher-level relationships with a rich sampling of ten nuclear and mitochondrial markers (7,726 bp) from 270 genera and find two distinct but equally plausible topologies among subfamilies at the base of the tree. In one set of analyses, the nuclear markers suggest two contrasting topologies, one of which is supported by the mitochondrial dataset. However, another set of analyses suggests mito-nuclear conflict as the reason for topological incongruence. Neither topology is strongly supported, and we conclude that there is insufficient phylogenetic evidence in the molecular dataset to resolve these relationships. Nevertheless, taking morphological characters into consideration, we suggest that one of the topologies is more likely.

## Introduction

A robust phylogeny is the key to understanding historical macroevolutionary processes that have shaped extant diversity. For instance, a phylogenetic hypothesis is needed to address questions regarding patterns of morphological evolution, coevolution, and historical biogeography, as well as for a higher-level classification system. Among invertebrates, butterflies have been the most popular study systems in evolutionary biology (Boggs, Watt & Ehrlich, 2003). Relationships among and within butterfly families have been largely studied by phylogenetic analyses of DNA sequence data (Campbell, Brower & Pierce, 2000; Caterino et al., 2001; Braby, Vila & Pierce, 2006; Nazari, Zakharov & Sperling, 2007; Wahlberg et al., 2009; Simonsen et al., 2011; Heikkilä et al., 2012; Wahlberg et al., 2014; Espeland et al., 2015). Yet, the higher-level relationships among skipper butterflies, with more than 4,000 species in about 567 genera (Warren, Ogawa & Brower, 2008) and representing a fifth of the world’s butterfly fauna (Hernández-Roldán et al., 2014), are still unsatisfactorily resolved.

Until recently, the higher-level classification of the family that has been generally followed was that proposed by Evans (1949) based on morphological characters. However, a major problem with skipper systematics is the remarkable uniformity of morphological structure among skipper taxa, which makes phenotype-based grouping extremely challenging (Voss, 1952; Warren, Ogawa & Brower, 2008). Following multiple attempts over the last several decades (Voss, 1952; Ackery, 1984; Scott, 1985; Scott & Wright, 1990; Chou, 1994; Chou, 1999; Mielke, 2005), a recent study employing molecular data suggested a classification that included five subfamilies (Warren, Ogawa & Brower, 2008). This classification relied on analyses of one mitochondrial and two nuclear markers, a dataset of 2,085 bp. A subsequent analysis that added morphological data (49 characters) to the same molecular data led to a revised classification that included seven subfamilies (Warren, Ogawa & Brower, 2009).

Warren, Ogawa & Brower (2008, 2009) used Maximum Parsimony analyses with nodal support estimated through Bremer Support values (Bremer, 1994). Despite some strongly supported monophyletic taxa being recovered, many putative higher clades were unresolved. Specifically, uncertainty remained about relationships among the major clades within the subfamily Pyrginae. Furthermore, support for relationships among the monophyletic subfamilies Heteropterinae, Trapezitinae and Hesperiinae was weak to moderate. The status of Euschemoninae as sister to rest of the family, except Coeliadinae, received very low nodal support (Bremer support = 1), although this placement is corroborated by the early developmental characters of Euschemoninae, which are similar to those of Coeliadinae and Eudaminae (Warren, Ogawa & Brower, 2009).

Yuan et al. (2015) investigated relationships among a small subset of hesperiid taxa—23 genera from China—using 1,458 bp of mitochondrial sequence data. Their Maximum Likelihood (ML) tree also indicated uncertainty in the position of Eudaminae and Pyrginae. Another study based on complete mitochondrial genomes of six skipper butterflies representing five subfamilies (sensu Warren, Ogawa & Brower, 2009) failed to support the monophyly of Pyrginae (Kim et al., 2014).

In summary, existing studies on the higher-level relationships within this speciose butterfly family have indicated significant conflicts (Warren, Ogawa & Brower, 2008; Warren, Ogawa & Brower, 2009; Kim et al., 2014; Yuan et al., 2015), and we currently lack a robust higher level phylogenetic hypothesis for evolutionary studies or a subfamily-level classification. The reasons for conflicting topologies across studies and poor nodal support could be (a) incongruence among gene trees due to incomplete lineage sorting (Pollard et al., 2006; Whitfield & Lockhart, 2007), (b) ancestral introgression (Eckert & Carstens, 2008), (c) differences in characteristics of the datasets used (Nabholz et al., 2011), (d) inadequate taxon sampling, (e) insufficient data to resolve deeper nodes (Wolf et al., 2002; Rokas et al., 2003a) or, (f) a near-hard polytomy due to a rapid radiation (Kodandaramaiah et al., 2010).

In order to bring further understanding to the higher-level phylogeny of skipper butterflies, we assembled sequences of 10 gene regions from 270 genera and analyzed a 7,726 bp dataset using both parsimony and model-based tree reconstruction methods. We also compiled the complete mitochondrial genome of 15 skipper species across five subfamilies from GenBank to compare the tree from the mitochondrial genome with that of single mitochondrial and combined nuclear genes. Consistent with the existing conflict across studies (Warren, Ogawa & Brower, 2008; Warren, Ogawa & Brower, 2009; Kim et al., 2014; Yuan et al., 2015), our analyses showed conflicting topologies at the deeper nodes of the phylogeny. To understand the reasons for the uncertainty in the phylogenetic estimation, we followed an integrative approach with systematic data encoding and tree comparison.

## Methods

### Taxon and gene sampling

Our analyses were based on 311 ingroup specimens representing 270 hesperiid genera and 12 outgroup taxa (five Papilionidae, two Hedylidae and five Pieridae). This dataset builds on the previous study by Warren, Ogawa & Brower (2009) that included sequences of three protein-coding genes (mtDNA COI, EF1a and wingless). We sequenced an additional part of COI and seven more genes (ArgKin, CAD, GAPDH, IDH, MDH, RpS2 and RpS5) using protocols and primers from Wahlberg & Wheat (2008). A new primer pair was designed (Table S1) to amplify the gene IDH for certain taxa. We have also included 96 additional specimens representing 71 genera to the present analyses. Our taxon sampling accounts for 60–70% of the genera of Coeliadinae (six genera), Eudaminae (38 genera), Heteropterinae (seven genera), and Trapezitinae (11 genera); 40–50% of Pyrginae (73 genera) and Hesperiinae (134 genera), and 100% of Euschemoninae (one genus). The sequences for outgroups were acquired from GenBank. We included the morphological, behavioral and ecological data matrix used in Warren, Ogawa & Brower (2009) in certain analyses. The sequences generated during this study were deposited in GenBank. The molecular data matrix in our study comprised 7,726 characters, more than three times that of the previous dataset (Warren, Ogawa & Brower, 2008; Warren, Ogawa & Brower, 2009).

### Dataset encoding

Along with the analysis of the concatenated dataset (nt_123), we generated context specific datasets from the concatenated gene matrix for various analyses designed to identify potential sources of conflict and/or poor nodal support. In one analysis, accounting for the impact of compositional heterogeneity, we assigned ambiguity to all the sites that potentially experience synonymous change (degen_1) (Regier et al., 2010; Zwick, Regier & Zwickl, 2012). We also checked the extent of substitution saturation in each gene matrix using DAMBE v6.4.20 (Xia, 2013), which showed saturation in 3rd codon positions (Fig. S1). To account for substitution saturation and degeneracy, we removed the 3rd codon positions and the 1st codon positions coding for Arginine or Lysine (noLRall1 + nt2) (Regier et al., 2008). In further analyses, we removed the 3rd coding positions from the concatenated dataset (nt_12) or only from the mitochondrial genes (nuclear_123 + CO_12).

We also analyzed all the nuclear genes together (nuclear_123) and reconstructed multi-gene and single-gene trees for comparison. In subsequent analyses, we also combined the morphological, ecological and behavioral characters from Warren, Ogawa & Brower (2009) with certain molecular datasets—nt_123 and nuclear_123. In addition, we analyzed an assembly of all protein coding sequences (13 genes) from the mitochondrial genomes of 15 skipper butterflies acquired from GenBank.

### Phylogenetic analyses

We performed Maximum Parsimony analyses in TNT v.1.1 (Goloboff, Farris & Nixon, 2008) using ‘New Technology’ searches (Goloboff, 1999; Nixon, 1999) (consisting of tree fusion, sectorial search, ratchet and tree drift) with 1,000 random addition replicates; nodal supports were derived from 1,000 bootstrap replicates (Felsenstein, 1985). For ML and Bayesian Inference (BI) analyses, we used RAxML v8 (Stamatakis, 2014) and MrBayes v3.2 (Ronquist et al., 2012), respectively, on the XSEDE web server through the CIPRES Science gateway (Miller, Pfeiffer & Schwartz, 2010). For ML analyses, we used the GTR model of substitution with gamma model of rate heterogeneity (GTR+G) and different partition schemes, either gene-based or based on rates of evolution calculated by the program Tree Independent Generation of Evolutionary Rates (TIGER) (Cummins & McInerney, 2011). In gene-based partitions each gene was considered as a separate partition, while in TIGER-based partitions, the characters were binned together based on their rate of evolution regardless of gene origin (used as a partitioning strategy in Rota & Wahlberg, 2012). TIGER partitions for the dataset were derived from the program TIGER v1.02 that calculates relative rates of evolution of each site in an alignment (Cummins & McInerney, 2011). The data were then divided into seven partitions based on the relative rates using an algorithm developed by Tobias Malm (J. Rota, T. Malm & N. Wahlberg, 2016, unpublished data) such that the first partition consisted of the invariant and very slowly evolving sites and the last partition consisted of sites evolving very quickly. To check whether model selection had any impact on the tree reconstruction, we also performed the above ML analyses using the best fitted model from PartitionFinder v1.1.1 (Lanfear et al., 2012) (detail in Fig. S2). For each ML analyses, the node supports were computed from 1,000 bootstrap replicates. Single-gene matrices were analyzed with GTR+G model and the node supports were derived from 500 bootstrap replicates. For single-gene analyses, we dropped the taxa for which the corresponding gene sequence was not available. For the mitogenome analysis, we performed ML tree searches with codon-based partitions and estimated the nodal support from 100 bootstraps.

The BI analysis of the concatenated dataset with TIGER partitions was performed with a mixed model of substitution which samples all possible models in the GTR family in proportion to their posterior distributions (Huelsenbeck, Larget & Alfaro, 2004) as implemented in MrBayes 3.2 (Ronquist et al., 2012). We assigned the gamma model of rate heterogeneity to all the partitions; the first partition was additionally assigned a proportion of invariable sites. The program MrBayes was set to estimate the base frequencies and shape parameters from the data. Two independent runs with two chains per run were performed for ∼30 million generations, sampling trees every 10,000 generations. The convergence of independent runs was analyzed from the values of potential scale reduction factors (PSRF) (PSRF close to one determines convergence) (Gelman & Rubin, 1992); we also checked the plots of log-likelihoods and other parameters on Tracer v1.6 (Rambaut et al., 2014).

### Tree comparison

To investigate the differences among the trees from multiple analyses, we compared the trees (except single-gene trees) for their topological incongruence with and without the likelihood scores. While the non likelihood-based incongruence test reflects differences in branching patterns among the topologies, the likelihood-based comparison calculates the difference between competing hypotheses with distinct topologies for a given dataset (Planet, 2006). For the non likelihood-based tree comparison at the deep level divergences, we used a Lento plot to depict the conflict among different trees in a two-dimensional graph (Lento et al., 1995). We employed the approximate unbiased (AU) test (Shimodaira, 2002) for likelihood-based tree comparisons. To check for incongruence among gene trees, we used partitioned bremer support (PBS) analysis that examines the contribution of each gene partition to the topological support of the consensus tree (Baker & DeSalle, 1997).

## Results

### Multilocus tree estimation

**Figure 1:**
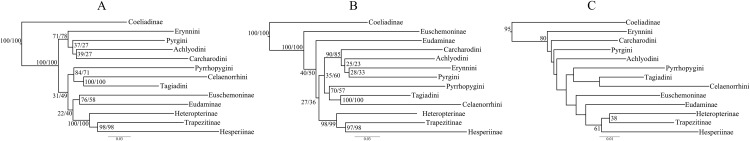
Comparison of best ML trees across analyses. The best ML trees from (A) the concatenated dataset, (B) combined nuclear dataset and (C) degenerated dataset (degen_1). The values at nodes in (A) and (B) represent support from 1,000 bootstrap trees analyzed with TIGER partitions/gene partitions. In (C), the values at the nodes are the output from the analyses using TIGER partitions only and the nodes without any value have bootstrap support <20. The other degenerated datasets (nt_12, noLRall1 + nt2) have similar or more fluctuating rearrangements for the nodes with BS < 20.

Trees from the concatenated dataset, irrespective of parsimony or ML analysis or the partition scheme used, showed identical relationships among the early branches representing the major clades (sensu Warren, Ogawa & Brower, 2009) (Fig. 1A)—(i) Coeliadinae was sister to the rest of the family, (ii) Pyrginae was paraphyletic, (iii) Euschemoninae was sister to Eudaminae, and (iv) Heteropterinae, Trapezitinae and Hesperiinae were all monophyletic. The topology remained unchanged even after the addition of morphological characters to the concatenated dataset.

However, the ML trees from the combined nuclear dataset (nuclear_123) showed a contrasting topology (Fig. 1B) to that of the concatenated dataset, irrespective of the partitioning scheme: Pyrginae was monophyletic and Euschemoninae was sister to rest of Hesperiidae except Coeliadinae. The topology remained unchanged with the addition of morphological characters to the dataset (nuclear_123 + morph) as well as analyses of the subsets of the combined nuclear matrix (7gene_123, 8gene_123).

These dataset-specific variations in tree topologies were consistent across different evolutionary models and also found when partition schemes from PartitionFinder were used (detail in Fig. S2).

ML analyses of non-degenerated datasets (nt_12, degen_1, noLRall1 + nt2) resulted in unresolved tree topologies indicating insufficient phylogenetic signal in the 1st and 2nd codon positions of the dataset (Fig. 1C).

BI analysis of the concatenated dataset showed a topology similar to that of Fig. 1B with a few changes (detail in Fig. S3). Randomly sampled 100 trees from the MCMC generations, after discarding burnin, showed the presence of only one topology (as in Fig. 1B); however, a very low proportion (6%) of an alternate topology (as in Fig. 1A) was found in one of the runs.

### Tree comparison

To test whether multiple tree topologies across ML analyses are equally likely given the datasets, we performed an AU test (Shimodaira, 2002) for both the concatenated and combined nuclear datasets independently. The AU test, without any partitioning scheme, rejected (p < 0.0001) the tree topologies from the non-degenerate datasets (nt_12, degen_1, noLRall1 + nt_2). Hence, we dropped the trees from non-degenerate data sets from further analyses of tree topological similarity at higher taxonomic levels. However, two distinct topologies (Figs. 1A and 1B) were accepted as significant trees (p > 0.05) for the combined nuclear dataset, whereas only one topology (Fig. 1A) was significant (p > 0.05) for the concatenated dataset.

A visual comparison of the two distinct tree topologies (Figs. 1A and 1B) showed that all the subfamily clades (sensu Warren, Ogawa & Brower, 2009) except Pyrginae were monophyletic (BS > 98). Pyrginae was recovered either as monophyletic (BS = 7–60) or paraphyletic. Similarly, Euschemoninae and Eudaminae were either sisters (BS = 58–76) or non-sisters. However, due to the presence of low BS values (<76) in six out of 13 deep nodes across the analyses, we were uncertain whether there existed significant conflict among different tree topologies.

### Mito-genomic analysis

The ML tree from all 13 protein coding genes from complete mitochondrial genomes of 15 skipper butterflies had a tree-wide average BS of 80 (see Fig. S4). Along with many well-supported nodes (BS > 97), low support values (BS 30–60) were also obtained for five out of 14 internal nodes. The lowest BS (= 32) was obtained for the node containing Eudaminae, the lineages of Pyrginae and the common ancestor of Heteropterinae and Hesperiinae. The node that placed Eudaminae as sister to one of the Pyrginae clades had BS = 42.

We found that the tree topology from the mitogenomic analysis corroborated the deep splits found in the COI gene tree; Eudaminae nested within Pyrginae rendering the latter paraphyletic, which was also the case for the concatenated dataset.

### Multiple plausible tree topologies

The presence of multiple tree topologies was not limited to the dataset-specific analyses. Regardless of the partitioning scheme or the dataset (concatenated and combined nuclear dataset) used, almost equal numbers of contrasting tree topologies were present among the ML-bootstrap trees across multiple analyses (see Fig. S5); hence, it is likely that the topology of the best ML tree is one among two equally likely topologies.

**Figure 2:**
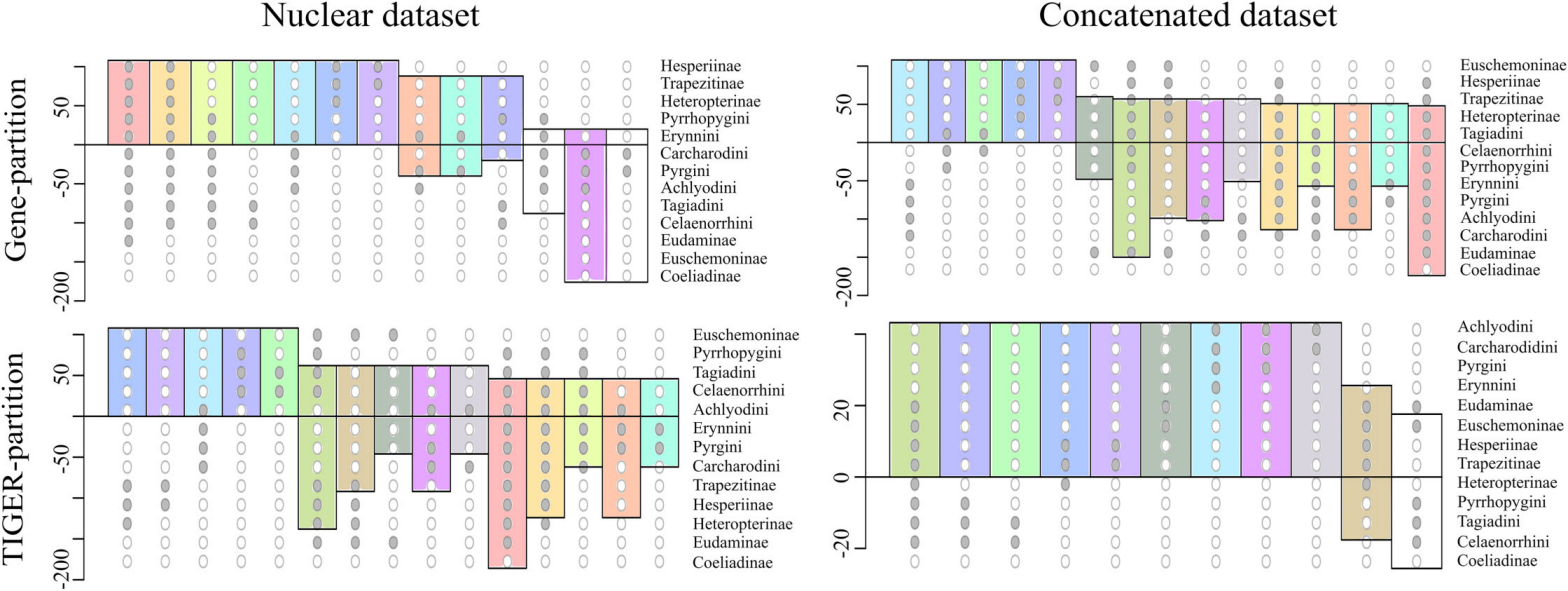
Comparison of the supports/conflicts on the Lento plots. The Lento plots were drawn from 105 ML trees recovered during best ML tree search across different datasets using two different partitioning schemes. The X-axis represents each non-trivial clade, with filled circles indicating the clade composition; the Y-axis shows relative support (values above zero) or conflict (values below zero). Splits are colour coded to aid comparison across analyses. The splits which are not coloured are found only in that particular analysis and not recovered from others.

To investigate this further, we extracted 105 model-optimized trees from each of the ML analyses of the concatenated (nt_123) and combined nuclear (nuclear_123) data, separately from datasets with both partitioning schemes (gene partitions and TIGER partitions). The clades in the resulting trees were collapsed to the subfamily level except Pyrginae, which was collapsed to the level of tribes (sensu Warren, Ogawa & Brower, 2009), and were plotted on a Lento plot (Fig. 2) to check for support and conflict for each split. We observed conflicting splits among the trees from the combined nuclear dataset with TIGER partitions, which indicated the presence of multiple topologies; however, trees from the concatenated dataset had no conflicting splits, indicating a single topology. But the case was different when gene partitions were used—multiple topologies resulted from the concatenated dataset and only one of these topologies was recovered from the combined nuclear dataset. The AU test showed equal tree likelihoods (p > 0.05) for multiple topologies for both concatenated and combined nuclear datasets given the respective partition schemes as explained above.

Thus, we found two contrasting tree topologies, respectively supporting (i) monophyly of Pyrginae and non-sister status of Eudaminae and Euschemoninae, and (ii) paraphyly of Pyrginae and sister status of Eudaminae and Euschemoninae. In addition, we noticed that the position of Eudaminae with respect to Pyrginae varied across these contrasting topologies. While Eudaminae was sister to all Hesperiidae except Coeliadinae and Euschemoninae in the former, the latter topology indicated that Eudaminae was sister to the clade containing Heteropterinae, Trapezitinae and Hesperiinae.

We observed that the contrasting topologies had short branch lengths at the fluctuating clades: the interrelationship among Eudaminae, the major clades of Pyrginae and the common ancestor of Heteropterinae, Trapezitinae and Hesperiinae. The association of very low BS (<60) with these short branches suggests possible topological conflict among gene trees. To investigate this hypothesis, we examined the relationships among the conflicting clades in the individual gene trees. Because the single-gene trees had very low BS values for most of the clades, we clustered gene markers based on their topological congruency for the deeper clades and reanalyzed, expecting an improvement of nodal support and consistency in signal. For example, we combined all those gene markers from which Pyrginae was recovered as monophyletic and expected an improved BS for the Pyrginae clade in the gene cluster analysis. As expected, this gene cluster recovered Pyrginae as monophyletic with higher node support (BS = 80). However, none of the gene clusters recovered the non-sister status of Eudaminae and Euschemoninae even though their sister status was poorly supported in all the gene cluster analyses (Fig. S6). This pattern of incongruence is not an artifact of missing data in our dataset, because sequential removal of taxa with 80–40% of missing data from the analyses changed neither the topology nor the support values at deeper nodes (Table S2); however, such sequential removal gradually reduced the proportion of alternate tree topologies in the tree set from best ML tree search (Fig. S7). Hence, we could not accept the hypothesis that the two contrasting tree topologies are due to incongruence in gene histories; this is also evident from PBS analysis where no pattern of incongruence among gene trees were observed (Fig. S8).

## Discussion

With a dataset of 7,726 bp from 270 hesperiid genera, we present the most comprehensive phylogeny of this important group of butterflies. Our analyses suggested that there are two contrasting topologies for the higher-level skipper phylogeny. First, we reconstructed the phylogenetic trees using the concatenated and combined nuclear datasets; the resulting trees were well-supported for higher-level relationships except at certain deep nodes. Tree comparisons revealed that there are multiple tree topologies for the relationships among major skipper lineages. We explicitly investigated gene-specific signals for the relationships among major clades, clustered them based on their topological congruence and reanalyzed to check for consistency.

### Conflicting topologies?

Our analyses indicate the occurrence of two equally likely deep tree topologies (Figs. 1A and 1B). Interestingly, the proximate reasons for the occurrence of these contrasting topologies appear to vary depending on the partitioning scheme used for the analysis. However, neither topology was strongly supported in any analysis and the results from our explorations of incongruence among gene histories were not conclusive. Gene cluster analyses improved the nodal support for the monophyly of Pyrginae but were unable to recover the sister status of Eudaminae and Euschemoninae with good support. Similarly, from the PBS analysis, no pattern of incongruence in gene histories was observed. We conclude that there is insufficient information in the molecular dataset to resolve these relationships despite the extensive taxonomic sampling and large number of molecular characters.

The presence of conflicting topologies has also been reported from many other studies across plants (Soltis, Soltis & Zanis, 2002; Burleigh & Mathews, 2004; Ruhfel et al., 2014) and animals (Rokas et al., 2003a; Song et al., 2012). The possible reasons for topological incongruency are phylogenetic noise or conflict among gene trees (Smith et al., 2015). In case of the former, a concatenation approach is expected to give a better result (Rokas et al., 2003b; Smith et al., 2015). In the latter case, where the conflict is presumed to be a result of gene flow across taxa or incomplete lineage sorting, coalescence based methods have been used for tree reconstruction (Jarvis et al., 2014; Xi et al., 2014; Smith et al., 2015). However, there was very low node supports across gene trees, indicating that strong conflict across genes does not explain the patterns found here. We predict that a phylogenomic approach would provide a better outlook to this conflicting scenario or resolve the phylogeny, as such an approach has proved instrumental in other studies (Dunn et al., 2008; Kocot et al., 2011; Smith et al., 2011; Johnson et al., 2013; Jarvis et al., 2014; Richart, Hayashi & Hedin, 2016).

### Systematic implications

**Figure 3:**
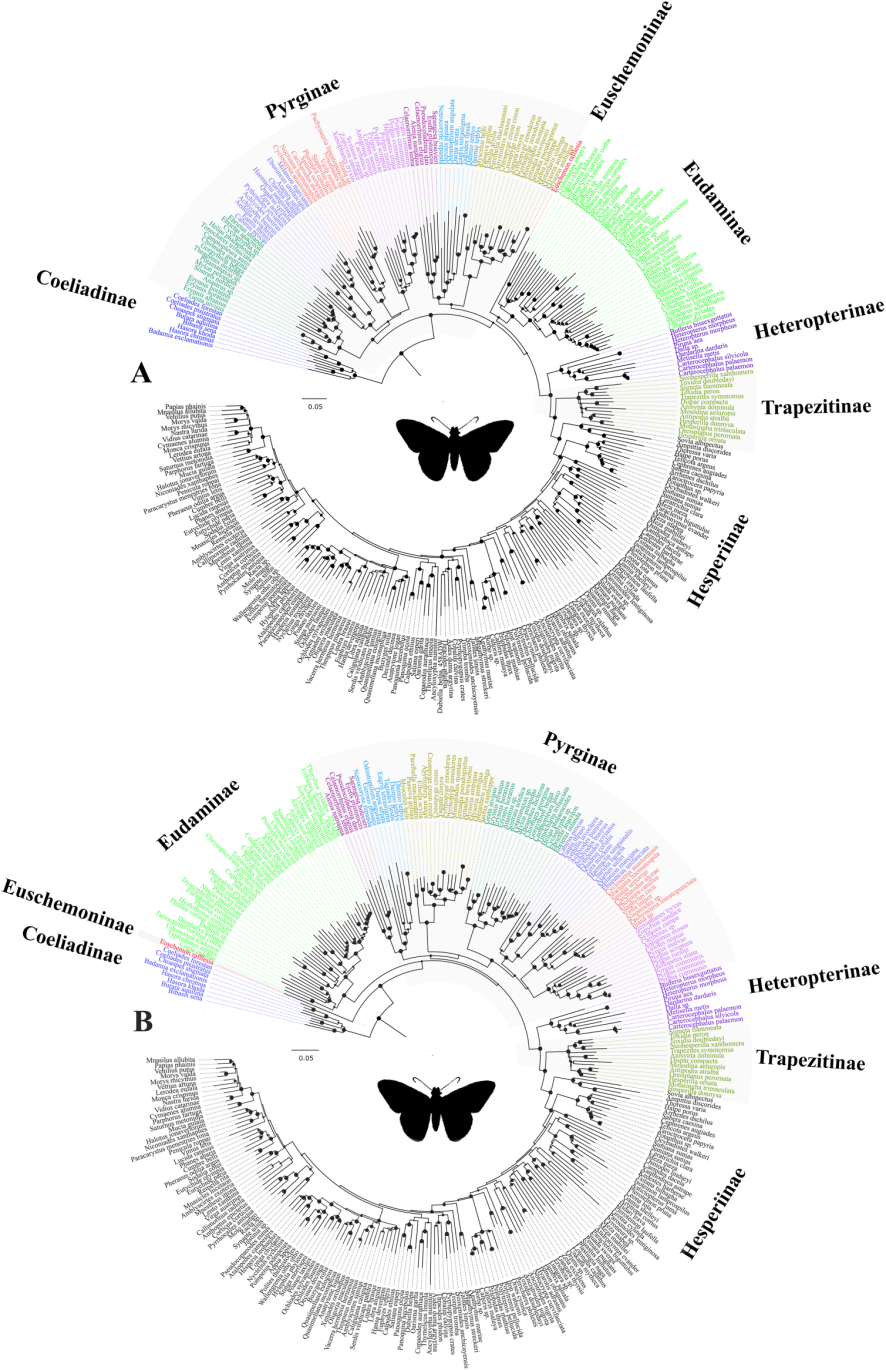
The ML trees from a reduced dataset. The ML trees from the analyses of (A) the concatenated dataset with gene partitions, and (B) the combined nuclear dataset with gene partitions. *Clito*, *Eracon* and three additional taxa were removed prior to the analyses (see text for detail). The size of the circle at the node corresponds to the bootstrap support that was derived from 1,000 pseudo-replicates. All taxa are colour coded based on their subfamily status, except the taxa within subfamily Pyrginae which are coloured based on their tribe. Silhouette from PhyloPic (http://phylopic.org/).

Our study confirmed that all the subfamilies, possibly except Pyrginae, are monophyletic and received high BS support across multiple analyses. We are uncertain about the monophyly of Pyrginae, as our study reveals homoplastic character distributions that could potentially be explained by the occurrence of ancestral introgression among its early lineages. Hence, when a certain combination of genes was used for phylogenetic construction, Pyrginae was recovered monophyletic (e.g., Fig. 1B), as in the previous study (Warren, Ogawa & Brower, 2009). This result appears to be supported by morphology. Similarly, we remain uncertain about the true relationships among Eudaminae, Pyrginae and the clade containing Heteropterinae, Trapezitinae and Hesperiinae, due to short branches that may be explained by their rapid divergence from each other and possibly an introgression between Eudaminae and Euschemoninae. However, the arrangement of Euschemoninae as sister to all Hesperiidae (except Coeliadinae), and Eudaminae as sister to Pyrginae, is generally supported by morphology. We suggest that the relationships shown in Fig. 1B (also in Fig. 3B) should be used as the preferred phylogenetic hypothesis until a better-resolved phylogeny is available.

In addition, we observed unexpected placement of a few taxa within Pyrginae. For instance, *Eracon*, which was previously classified under Pyrgini (Warren, Ogawa & Brower, 2009), was found herein to group with Achlyodini. Likewise, *Clito* grouped within either Pyrgini or Erynnini based on dataset specific analyses. Moreover, we note that both *Clito* and *Eracon* sequences in our dataset have >70% missing sites. Hence, it is likely that the presence of insufficient informative sites within these taxa might influence their true positions in the phylogeny (Wiens, 2003; Wiens & Morrill, 2011). Therefore, for systematic implications, we pruned *Clito*, *Eracon* and three additional taxa with a large percentage of missing data from the dataset and reanalysed with gene partitions (Fig. 3). We observed no change in tree topology or node support values as a result of pruning these taxa. Hence, although they may appear on the tree in unorthodox positions, it is unlikely that presence of these taxa has any impact on our interpretation of higher-level relationships in the dataset as a whole.

We observed that the genus *Cabirus*, previously included within Eudaminae, grouped within Achlyodini (subfamily Pyrginae). Further study of the morphology of *Cabirus* is needed to corroborate this placement, although its position outside of Eudaminae seems to be correct. Three tribes within Hesperiinae—Aeromachini, Taractrocerini and Baorini—are monophyletic with high BS values. However, we are uncertain about the phylogenetic status of other proposed tribes within Hesperiinae due to prevalence of low BS values along the short internal branches. This indicates the possible occurrences of rapid ancestral radiation within Hesperiinae and needs further investigation.

## Conclusions

With a broad coverage of all known subfamilies, we present the higher-level relationships among skipper butterflies. Our analyses suggest possible conflicting topologies with respect to (i) monophyly or paraphyly of Pyrginae and (ii) sister or non-sister status of Eudaminae and Euschemoninae. However, none of the topologies resulting from our alternative analyses is strongly supported, and incongruences in signal among genes cannot satisfactorily resolve these differences. We surmise that there is insufficient phylogenetic information in the current dataset to resolve these relationships. It is unlikely that adding data from a few more genes will improve the results, but data from entire genomes may result in a better-resolved phylogeny. However, taking morphological characters into consideration, we suggest one of the topologies as most likely (Figs. 1B and 3B), and that this topology will aid in future studies on this group.

## Supplemental Information

10.7717/peerj.2653/supp-1 Supplemental Information 1 Newly designed primer pairs for an IDH (Isocitrate dehydrogenase) amplicon in Hesperiidae. This primer pair, along with attached universal tail (T7 promoter and T3) were tested at annealing temperature 55 °C.

10.7717/peerj.2653/supp-2 Supplemental Information 2 Summarization of tree properties at different proportion of missing data. The bootstrap support (BS) and branch length for the deep nodes from the ML trees reconstructed using gene partitions for different datasets. The dataset1 is same as the concatenated dataset (> 80% missing data per taxa allowed) and the other datasets were generated after removal of taxa with more than the maximum proportion of permitted missing sites. For instance, to generate dataset6, we removed all those taxa which had more than 40% missing sites.

10.7717/peerj.2653/supp-3 Supplemental Information 3 New taxonomic positions of few genera. List of genera for which we observed new taxonomic positions or ambiguity in taxonomic position. The taxonomic positions of rest of the sampled genera follow the list supplied with Warren, Ogawa & Brower (2009).

10.7717/peerj.2653/supp-4 Supplemental Information 4 Saturation curve of 3rd codon positions on F84 sequence distance. Transitions: s; blue cross and transversions: v; green triangle. For all genes, the curve saturate at higher sequence divergence or when the genetic distances between sequences increase. (The saturation curves for CAD and GAPDH are not shown because of consistent technical error during analyses which may be due to high sequence divergences within these gene sets crosses the limit of permitted scale length by the software.).

10.7717/peerj.2653/supp-5 Supplemental Information 5 ML trees from concatenated and combined nuclear dataset under different partition scheme or model of evolution. ML trees from the analyses of concatenated dataset with the partitions from PartitionFinder (A) and with TIGER partitions (B). ML trees from the analyses of combined nuclear dataset with the partitions from PartitionFinder (C) and with TIGER partitions (D). GTR+G+I model was used for all the analyses and the node supports were calculated from 1,000 bootstraps.

10.7717/peerj.2653/supp-6 Supplemental Information 6 Bayesian tree from concatenated dataset. The analysis was performed on the concatenated dataset with TIGER partitions under reversible jump MCMC. The values at nodes represent posterior probabilities. The topology in this tree is similar to that of Fig. 1B except that Euschemoninae and Eudaminae are sisters.

10.7717/peerj.2653/supp-7 Supplemental Information 7 The maximum likelihood tree from mitogenome analysis. The maximum likelihood tree from the analyses of 13 protein-coding mitochondrial genes of 6 hesperiids using codon-based partitions. The node supports were derived from 100 bootstrap analysis.

10.7717/peerj.2653/supp-8 Supplemental Information 8 Cross representation of bootstrap support. (A) Summarization of the ML-bootstrap trees from the concatenated dataset on the ML-best tree from the combined nuclear dataset. (B) Summarization of the ML-bootstrap trees from the combined nuclear dataset on the ML-best tree from the concatenated dataset.

10.7717/peerj.2653/supp-9 Supplemental Information 9 Analyses of gene clusters. Trees from the ML analyses of different gene combinations (A) ArgKin, EF1a, GAPDH, RpS2, RpS5, wingless, (B) CAD, EF1a, IDH, wingless, (C) ArgKin, GAPDH, MDH, RpS2, RpS5 and (D) CAD, MDH using gene partitions. The node supports were derived from 1,000 bootstrap trees. The gene combinations were made based on the relationships recovered in single-gene trees for clade status of Pyrginae and sister status of Eudaminae and Euschemoninae. Thus, the expected relationships in: (A) is that Eudaminae and Euschemoninae are non-sisters, (B) Pyrginae is monophyletic, (C) Pyrginae is paraphyletic and (D) Eudaminae and Euschemoninae are sisters.

10.7717/peerj.2653/supp-10 Supplemental Information 10 Proportion of contrasting tree topologies across datasets. The proportion of trees, that show either topology 1 (as in Fig. 1A) or topology 2 (as in Fig. 1B), in the set of 105 or 108 independent ML trees from the analyses of different datasets as mentioned in Table S2. The number on the top of the bar represents the number of ingroup taxa in the respective dataset.

10.7717/peerj.2653/supp-11 Supplemental Information 11 Summarization of partitioned bremer support. (A) The node specific partitioned bremer support for ten-gene partitions. (B) The positions of the nodes are shown on the consensus tree reconstructed using concatenated dataset.

10.7717/peerj.2653/supp-12 Supplemental Information 12 Concatenated aligned sequence file. The gene partitions in this concatenated aligned sequence file is: ArgKin = 1-596, CAD = 597-1446, COI = 1447-2921, EF1A = 2922-416, GAPDH = 4162-4852, IDH = 4853-5562, MDH = 5563-6298, RPS2 = 6299-6709, RPS5 = 6710-7326, WGL = 7327-7726.

10.7717/peerj.2653/supp-13 Supplemental Information 13 List of sequences of length less than 200 bp in fasta format. The definition of each sequence include the name of organism, the specimen voucher, gene name, sequence length and the starting position of codon.

## Additional Information and Declarations

### DNA Deposition

The following information was supplied regarding the deposition of DNA sequences: GenBank: KX947011–KX947012, KX947014–KX947266, KY014330–KY014419, KY019648–KY020025, KY027462–KY028742, KY045507–KY045764.

### Data Deposition

The following information was supplied regarding data availability: The raw data has been supplied as Supplemental Dataset Files.

## References

[ref-1] Ackery PR, Vane-Wright RI, Ackery PR (1984). Systematic and faunistic studies on butterflies. The Biology of Butterflies.

[ref-2] Baker RH, DeSalle R (1997). Multiple sources of character information and the phylogeny of Hawaiian drosophilids. Systematic Biology.

[ref-3] Boggs CL, Watt WB, Ehrlich PR (2003). Butterflies: Ecology and Evolution Taking Flight.

[ref-4] Braby MF, Vila R, Pierce NE (2006). Molecular phylogeny and systematics of the Pieridae (Lepidoptera: Papilionoidea): higher classification and biogeography. Zoological Journal of the Linnean Society.

[ref-5] Bremer K (1994). Branch support and tree stability. Cladistics.

[ref-6] Burleigh JG, Mathews S (2004). Phylogenetic signal in nucleotide data from seed plants: implications for resolving the seed plant tree of life. American Journal of Botany.

[ref-7] Campbell DL, Brower AVZ, Pierce NE (2000). Molecular evolution of the *wingless* gene and its implications for the phylogenetic placement of the butterfly family Riodinidae (Lepidoptera: Papilionoidea). Molecular Biology and Evolution.

[ref-8] Caterino MS, Reed RD, Kuo MM, Sperling FAH (2001). A partitioned likelihood analysis of swallowtail butterfly phylogeny (Lepidoptera: Papilionidae). Systematic Biology.

[ref-9] Chou I (1994). Monographia Rhopalocerorum Sinensium.

[ref-10] Chou I (1999). Classification and Identification of Chinese Butterflies.

[ref-11] Cummins CA, McInerney JO (2011). A method for inferring the rate of evolution of homologous characters that can potentially improve phylogenetic inference, resolve deep divergence and correct systematic biases. Systematic Biology.

[ref-12] Dunn CW, Hejnol A, Matus DQ, Pang K, Browne WE, Smith SA, Seaver E, Rouse GW, Obst M, Edgecombe GD, Sørensen MV, Haddock SHD, Schmidt-Rhaesa A, Okusu A, Kristensen RM, Wheeler WC, Martindale MQ, Giribet G (2008). Broad phylogenomic sampling improves resolution of the animal tree of life. Nature.

[ref-13] Eckert AJ, Carstens BC (2008). Does gene flow destroy phylogenetic signal? The performance of three methods for estimating species phylogenies in the presence of gene flow. Molecular Phylogenetics and Evolution.

[ref-14] Espeland M, Hall JPW, DeVries PJ, Lees DC, Cornwall M, Hsu Y-F, Wu L-W, Campbell DL, Talavera G, Vila R, Salzman S, Ruehr S, Lohman DJ, Pierce NE (2015). Ancient Neotropical origin and recent recolonisation: phylogeny, biogeography and diversification of the Riodinidae (Lepidoptera: Papilionoidea). Molecular Phylogenetics and Evolution.

[ref-15] Evans WH (1949). A Catalogue of the Hesperiidae from Europe, Asia, and Australia in the British Museum (Natural History).

[ref-16] Felsenstein J (1985). Phylogenies and the comparative method. The American Naturalist.

[ref-17] Gelman A, Rubin DB (1992). Inference from iterative simulation using multiple sequences. Statistical Science.

[ref-18] Goloboff PA (1999). Analyzing large data sets in reasonable times: solutions for composite optima. Cladistics.

[ref-19] Goloboff PA, Farris JS, Nixon KC (2008). TNT, a free program for phylogenetic analysis. Cladistics.

[ref-20] Heikkilä M, Kaila L, Mutanen M, Peña C, Wahlberg N (2012). Cretaceous origin and repeated tertiary diversification of the redefined butterflies. Proceedings of the Royal Society B: Biological Sciences.

[ref-21] Hernández-Roldán JL, Bofill R, Dapporto L, Munguira ML, Vila R (2014). Morphological and chemical analysis of male scent organs in the butterfly genus Pyrgus (Lepidoptera: Hesperiidae). Organisms Diversity & Evolution.

[ref-22] Huelsenbeck JP, Larget B, Alfaro ME (2004). Bayesian phylogenetic model selection using reversible jump markov chain monte carlo. Molecular Biology and Evolution.

[ref-23] Jarvis ED, Mirarab S, Aberer AJ, Li B, Houde P, Li C, Ho SYW, Faircloth BC, Nabholz B, Howard JT, Suh A, Weber CC, da Fonseca RR, Li J, Zhang F, Li H, Zhou L, Narula N, Liu L, Ganapathy G, Boussau B, Bayzid MS, Zavidovych V, Subramanian S, Gabaldón T, Capella-Gutiérrez S, Huerta-Cepas J, Rekepalli B, Munch K, Schierup M, Lindow B, Warren WC, Ray D, Green RE, Bruford MW, Zhan X, Dixon A, Li S, Li N, Huang Y, Derryberry EP, Bertelsen MF, Sheldon FH, Brumfield RT, Mello CV, Lovell PV, Wirthlin M, Schneider MPC, Prosdocimi F, Samaniego JA, Velazquez AMV, Alfaro-Núñez A, Campos PF, Petersen B, Sicheritz-Ponten T, Pas A, Bailey T, Scofield P, Bunce M, Lambert DM, Zhou Q, Perelman P, Driskell AC, Shapiro B, Xiong Z, Zeng Y, Liu S, Li Z, Liu B, Wu K, Xiao J, Yinqi X, Zheng Q, Zhang Y, Yang H, Wang J, Smeds L, Rheindt FE, Braun M, Fjeldsa J, Orlando L, Barker FK, Jønsson KA, Johnson W, Koepfli K-P, O’Brien S, Haussler D, Ryder OA, Rahbek C, Willerslev E, Graves GR, Glenn TC, McCormack J, Burt D, Ellegren H, Alström P, Edwards SV, Stamatakis A, Mindell DP, Cracraft J, Braun EL, Warnow T, Jun W, Gilbert MTP, Zhang G (2014). Whole-genome analyses resolve early branches in the tree of life of modern birds. Science.

[ref-24] Johnson BR, Borowiec ML, Chiu JC, Lee EK, Atallah J, Ward PS (2013). Phylogenomics resolves evolutionary relationships among ants, bees, and wasps. Current Biology.

[ref-25] Kim MJ, Wang AR, Park JS, Kim I (2014). Complete mitochondrial genomes of five skippers (Lepidoptera: Hesperiidae) and phylogenetic reconstruction of Lepidoptera. Gene.

[ref-26] Kocot KM, Cannon JT, Todt C, Citarella MR, Kohn AB, Meyer A, Santos SR, Schander C, Moroz LL, Lieb B, Halanych KM (2011). Phylogenomics reveals deep molluscan relationships. Nature.

[ref-27] Kodandaramaiah U, Peña C, Braby MF, Grund R, Müller CJ, Nylin S, Wahlberg N (2010). Phylogenetics of Coenonymphina (Nymphalidae: Satyrinae) and the problem of rooting rapid radiations. Molecular Phylogenetics and Evolution.

[ref-28] Lanfear R, Calcott B, Ho SYW, Guindon S (2012). PartitionFinder: combined selection of partitioning schemes and substitution models for phylogenetic analyses. Molecular Biology and Evolution.

[ref-29] Lento GM, Hickson RE, Chambers GK, Penny D (1995). Use of spectral analysis to test hypotheses on the origin of pinnipeds. Molecular Biology and Evolution.

[ref-30] Mielke OHH (2005). Catalogue of the American Hesperioidea: Hesperiidae (Lepidoptera).

[ref-31] Miller MA, Pfeiffer W, Schwartz T (2010). Creating the CIPRES science gateway for inference of large phylogenetic trees.

[ref-32] Nabholz B, Kunstner A, Wang R, Jarvis ED, Ellegren H (2011). Dynamic evolution of base composition: causes and consequences in avian phylogenomics. Molecular Biology and Evolution.

[ref-33] Nazari V, Zakharov EV, Sperling FAH (2007). Phylogeny, historical biogeography, and taxonomic ranking of Parnassiinae (Lepidoptera, Papilionidae) based on morphology and seven genes. Molecular Phylogenetics and Evolution.

[ref-34] Nixon KC (1999). The parsimony ratchet, a new method for rapid parsimony analysis. Cladistics.

[ref-35] Planet PJ (2006). Tree disagreement: measuring and testing incongruence in phylogenies. Journal of Biomedical Informatics.

[ref-36] Pollard DA, Iyer VN, Moses AM, Eisen MB (2006). Widespread discordance of gene trees with species tree in Drosophila: evidence for incomplete lineage sorting. PLoS Genetics.

[ref-37] Rambaut A, Suchard MA, Xie D, Drummond AJ (2014). http://beast.bio.ed.ac.um/Tracer.

[ref-38] Regier JC, Shultz JW, Ganley AR, Hussey A, Shi D, Ball B, Zwick A, Stajich JE, Cummings MP, Martin JW, Cunningham CW (2008). Resolving arthropod phylogeny: exploring phylogenetic signal within 41 kb of protein-coding nuclear gene sequence. Systematic Biology.

[ref-39] Regier JC, Shultz JW, Zwick A, Hussey A, Ball B, Wetzer R, Martin JW, Cunningham CW (2010). Arthropod relationships revealed by phylogenomic analysis of nuclear protein-coding sequences. Nature.

[ref-40] Richart CH, Hayashi CY, Hedin M (2016). Phylogenomic analyses resolve an ancient trichotomy at the base of Ischyropsalidoidea (Arachnida, Opiliones) despite high levels of gene tree conflict and unequal minority resolution frequencies. Molecular Phylogenetics and Evolution.

[ref-41] Rokas A, King N, Finnerty J, Carroll SB (2003a). Conflicting phylogenetic signals at the base of the metazoan tree. Evolution and Development.

[ref-42] Rokas A, Williams BL, King N, Carroll SB (2003b). Genome-scale approaches to resolving incongruence in molecular phylogenies. Nature.

[ref-43] Ronquist F, Teslenko M, van der Mark P, Ayres DL, Darling A, Höhna S, Larget B, Liu L, Suchard MA, Huelsenbeck JP (2012). MrBayes 3.2: efficient Bayesian phylogenetic inference and model choice across a large model space. Systematic Biology.

[ref-44] Rota J, Wahlberg N (2012). Exploration of data partitioning in an eight-gene dataset: phylogeny of metalmark moths (Lepidoptera, Choreutidae). Zoologica Scripta.

[ref-45] Ruhfel BR, Gitzendanner MA, Soltis PS, Soltis DE, Burleigh JG (2014). From algae to angiosperms-inferring the phylogeny of green plants (*Viridiplantae*) from 360 plastid genomes. BMC Evolutionary Biology.

[ref-46] Scott JA (1985). The phylogeny of butterflies (Papilionoidea and Hesperioidea). Journal of Research on the Lepidoptera.

[ref-47] Scott JA, Wright DM, Kudrna O (1990). Butterfly phylogeny and fossils. Butterflies of Europe.

[ref-48] Shimodaira H (2002). An approximately unbiased test of phylogenetic tree selection. Systematic Biology.

[ref-49] Simonsen TJ, Zakharov EV, Djernaes M, Cotton AM, Vane-Wright RI, Sperling FAH (2011). Phylogenetics and divergence times of Papilioninae (Lepidoptera) with special reference to the enigmatic genera Teinopalpus and Meandrusa. Cladistics.

[ref-50] Smith SA, Moore MJ, Brown JW, Yang Y (2015). Analysis of phylogenomic datasets reveals conflict, concordance, and gene duplications with examples from animals and plants. BMC Evolutionary Biology.

[ref-51] Smith SA, Wilson NG, Goetz FE, Feehery C, Andrade SCS, Rouse GW, Giribet G, Dunn CW (2011). Resolving the evolutionary relationships of molluscs with phylogenomic tools. Nature.

[ref-52] Soltis DE, Soltis PS, Zanis MJ (2002). Phylogeny of seed plants based on evidence from eight genes. American Journal of Botany.

[ref-53] Song S, Liu L, Edwards SV, Wu S (2012). Resolving conflict in eutherian mammal phylogeny using phylogenomics and the multispecies coalescent model. Proceedings of the National Academy of Sciences of the United States of America.

[ref-54] Stamatakis A (2014). RAxML version 8: a tool for phylogenetic analysis and post-analysis of large phylogenies. Bioinformatics.

[ref-55] Voss EG (1952). On the classification of the Hesperiidae. Annals of the Entomological Society of America.

[ref-56] Wahlberg N, Leneveu J, Kodandaramaiah U, Peña C, Nylin S, Freitas AVL, Brower AVZ (2009). Nymphalid butterflies diversify following near demise at the Cretaceous/Tertiary boundary. Proceedings of the Royal Society B: Biological Sciences.

[ref-57] Wahlberg N, Rota J, Braby MF, Pierce NE, Wheat CW (2014). Revised systematics and higher classification of pierid butterflies (Lepidoptera: Pieridae) based on molecular data. Zoologica Scripta.

[ref-58] Wahlberg N, Wheat CW (2008). Genomic outposts serve the phylogenomic pioneers: designing novel nuclear markers for genomic DNA extractions of Lepidoptera. Systematic Biology.

[ref-59] Warren AD, Ogawa JR, Brower AVZ (2008). Phylogenetic relationships of subfamilies and circumscription of tribes in the family Hesperiidae (Lepidoptera: Hesperioidea). Cladistics.

[ref-60] Warren AD, Ogawa JR, Brower AVZ (2009). Revised classification of the family Hesperiidae (Lepidoptera: Hesperioidea) based on combined molecular and morphological data. Systematic Entomology.

[ref-61] Whitfield JB, Lockhart PJ (2007). Deciphering ancient rapid radiations. Trends in Ecology & Evolution.

[ref-62] Wiens JJ (2003). Missing data, incomplete taxa, and phylogenetic accuracy. Systematic Biology.

[ref-63] Wiens JJ, Morrill MC (2011). Missing data in phylogenetic analysis: reconciling results from simulations and empirical data. Systematic Biology.

[ref-64] Wolf YI, Rogozin IB, Grishin NV, Koonin EV (2002). Genome trees and the tree of life. Trends in Genetics.

[ref-65] Xi Z, Liu L, Rest JS, Davis CC (2014). Coalescent versus concatenation methods and the placement of amborella as sister to water lilies. Systematic Biology.

[ref-66] Xia X (2013). DAMBE5: a comprehensive software package for data analysis in molecular biology and evolution. Molecular Biology and Evolution.

[ref-67] Yuan X, Gao K, Yuan F, Wang P, Zhang Y (2015). Phylogenetic relationships of subfamilies in the family Hesperiidae (Lepidoptera: Hesperioidea) from China. Scientific Reports.

[ref-68] Zwick A, Regier JC, Zwickl DJ (2012). Resolving discrepancy between nucleotides and amino acids in deep-level arthropod phylogenomics: differentiating serine codons in 21-amino acid models. PLoS ONE.

