# Supplementary material for: Ten genes and two topologies: an exploration of higher relationships in skipper butterflies (Hesperiidae)
Source: PeerJ. 2016 Dec 6;4:e2653. doi: 10.7717/peerj.2653 (PMC5144725; doi:10.7717/peerj.2653)
Supplement: Supplemental Information 1 — This primer pair, along with attached universal tail (T7 promoter and T3) were tested at annealing temperature 55 °C. [file peerj-04-2653-s001.docx]

| Name of Primer | Sequence (5’ – 3’) |
| --- | --- |
| IDH74F | CCGTGACAAAACTGAAGACCA |
| IDH691R | GCCACCATATCATCAATCAAGCG |
