# Supplementary material for: Ten genes and two topologies: an exploration of higher relationships in skipper butterflies (Hesperiidae)
Source: PeerJ. 2016 Dec 6;4:e2653. doi: 10.7717/peerj.2653 (PMC5144725; doi:10.7717/peerj.2653)
Supplement: Supplemental Information 2 — The bootstrap support (BS) and branch length for the deep nodes from the ML trees reconstructed using gene partitions for different datasets. The dataset1 is same as the concatenated dataset (> 80% missing data per taxa allowed) and the other datasets were generated after removal of taxa with more than the maximum proportion of permitted missing sites. For instance, to generate dataset6, we removed all those taxa which had more than 40% missing sites. [file peerj-04-2653-s002.docx]

|  | **Dataset 1** | | **Dataset 2** | | **Dataset 3** | | **Dataset 4** | | **Dataset 5** | | **Dataset 6** | |
| --- | --- | --- | --- | --- | --- | --- | --- | --- | --- | --- | --- | --- |
|  | **>80% missing sites per taxon allowed** | | **Max. 80% missing sites per taxon** | | **Max. 70% missing sites per taxon** | | **Max. 60% missing sites per taxon** | | **Max. 50% missing sites per taxon** | | **Max. 40% missing sites per taxon** | |
| Deep nodes | BS | Branch length | BS | Branch length | BS | Branch length | BS | Branch length | BS | Branch length | BS | Branch length |
| Node1 | 98 | 0.010 | 99 | 0.011 | 99 | 0.011 | 99 | 0.011 | 99 | 0.011 | 99 | 0.011 |
| Node2 | 100 | 0.022 | 100 | 0.022 | 100 | 0.022 | 100 | 0.022 | 100 | 0.022 | 100 | 0.021 |
| Node3 | 40 | 0.006 | 42 | 0.006 | 37 | 0.006 | 37 | 0.006 | 37 | 0.006 | 30 | 0.006 |
| Node4 | 58 | 0.008 | 59 | 0.008 | 58 | 0.009 | 57 | 0.008 | 58 | 0.009 | 55 | 0.009 |
| Node5 | 49 | 0.012 | 51 | 0.012 | 46 | 0.012 | 45 | 0.012 | 47 | 0.012 | 41 | 0.012 |
| Node6 | 100 | 0.048 | 100 | 0.048 | 100 | 0.049 | 100 | 0.047 | 100 | 0.048 | 100 | 0.044 |
| Node7 | 100 | 0.061 | 100 | 0.060 | 100 | 0.061 | 100 | 0.060 | 100 | 0.060 | 100 | 0.062 |
