# Supplementary material for: Ten genes and two topologies: an exploration of higher relationships in skipper butterflies (Hesperiidae)
Source: PeerJ. 2016 Dec 6;4:e2653. doi: 10.7717/peerj.2653 (PMC5144725; doi:10.7717/peerj.2653)
Supplement: Supplemental Information 3 — List of genera for which we observed new taxonomic positions or ambiguity in taxonomic position. The taxonomic positions of rest of the sampled genera follow the list supplied with Warren, Ogawa & Brower (2009). [file peerj-04-2653-s003.docx]

| Genera | Taxonomic position from Warren et al., 2009 | Taxonomic position from present analyses | Remarks |
| --- | --- | --- | --- |
| *Clito* | Pyrgini | Pyrgini or Erynnini | Such fluctuation in placement may due to >70% missing sites in the sequence of this taxa. |
| *Eracon* | Pyrgini | Achlyodini | We are not confident about this new placement as the sequence of this taxa has >70% missing sites. |
| *Cabirus* | Eudaminae | Achlyodini | Although its position outside Eudaminae seems correct, further study on morphology is necessary to corroborate this placement. |
