# Supplementary figures and images for: Ten genes and two topologies: an exploration of higher relationships in skipper butterflies (Hesperiidae)
Source: PeerJ. 2016 Dec 6;4:e2653. doi: 10.7717/peerj.2653 (PMC5144725; doi:10.7717/peerj.2653)

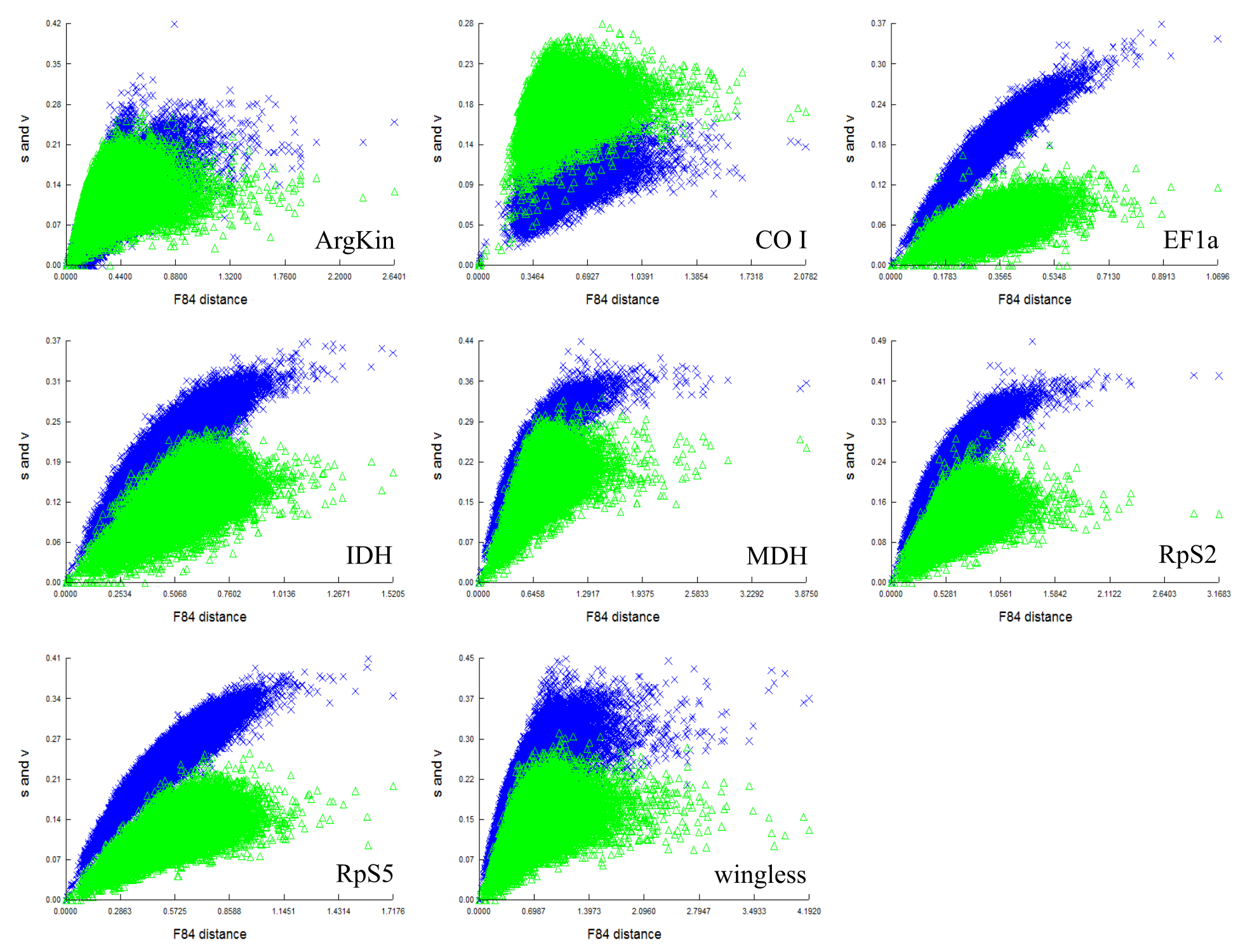

Supplement: Supplemental Information 4 — Transitions: s; blue cross and transversions: v; green triangle. For all genes, the curve saturate at higher sequence divergence or when the genetic distances between sequences increase. (The saturation curves for CAD and GAPDH are not shown because of consistent technical error during analyses which may be due to high sequence divergences within these gene sets crosses the limit of permitted scale length by the software.). [file peerj-04-2653-s004.png]

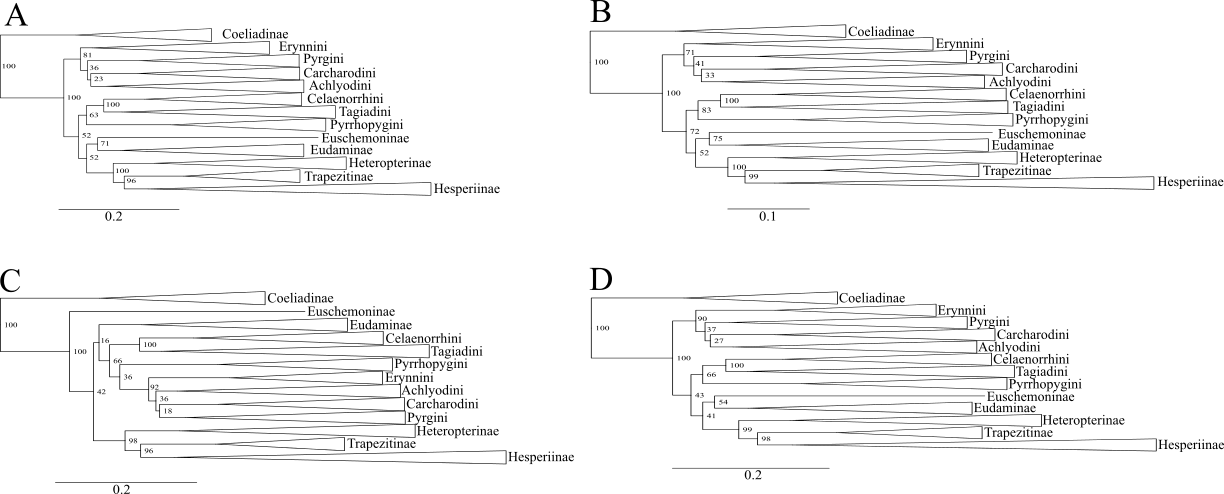

Supplement: Supplemental Information 5 — ML trees from the analyses of concatenated dataset with the partitions from PartitionFinder (A) and with TIGER partitions (B). ML trees from the analyses of combined nuclear dataset with the partitions from PartitionFinder (C) and with TIGER partitions (D). GTR+G+I model was used for all the analyses and the node supports were calculated from 1,000 bootstraps. [file peerj-04-2653-s005.png]

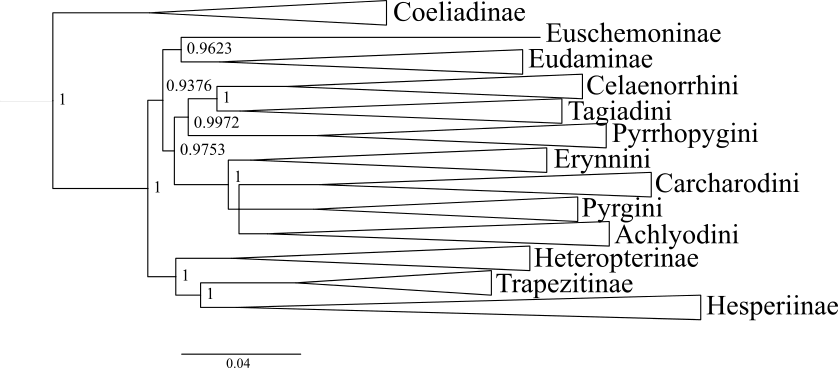

Supplement: Supplemental Information 6 — The analysis was performed on the concatenated dataset with TIGER partitions under reversible jump MCMC. The values at nodes represent posterior probabilities. The topology in this tree is similar to that of Fig. 1B except that Euschemoninae and Eudaminae are sisters. [file peerj-04-2653-s006.png]

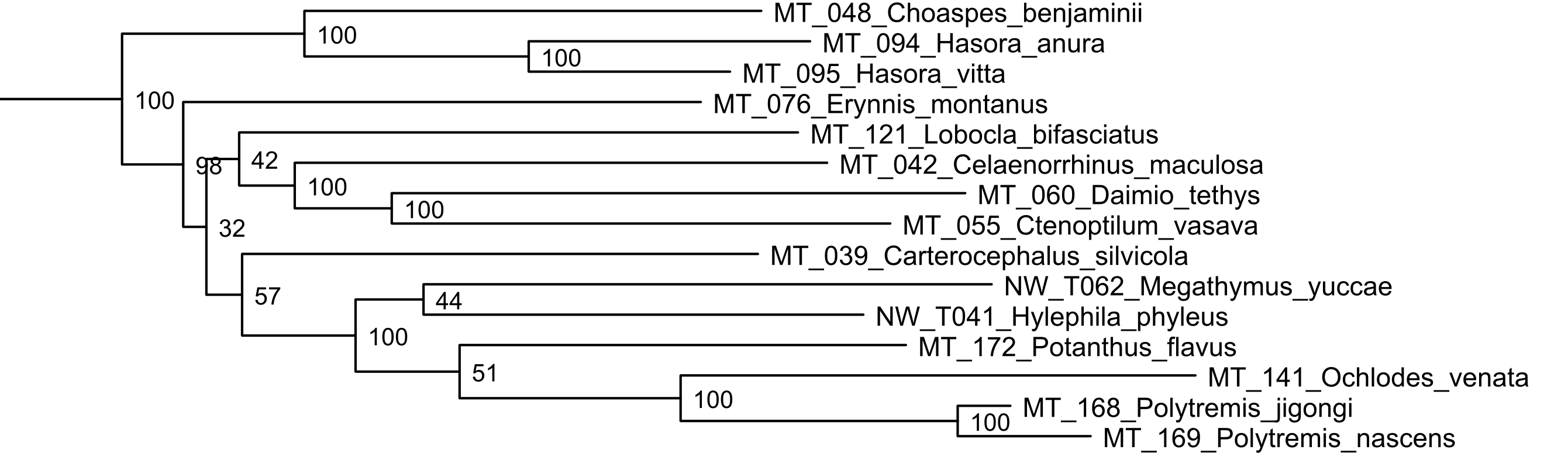

Supplement: Supplemental Information 7 — The maximum likelihood tree from the analyses of 13 protein-coding mitochondrial genes of 6 hesperiids using codon-based partitions. The node supports were derived from 100 bootstrap analysis. [file peerj-04-2653-s007.png]

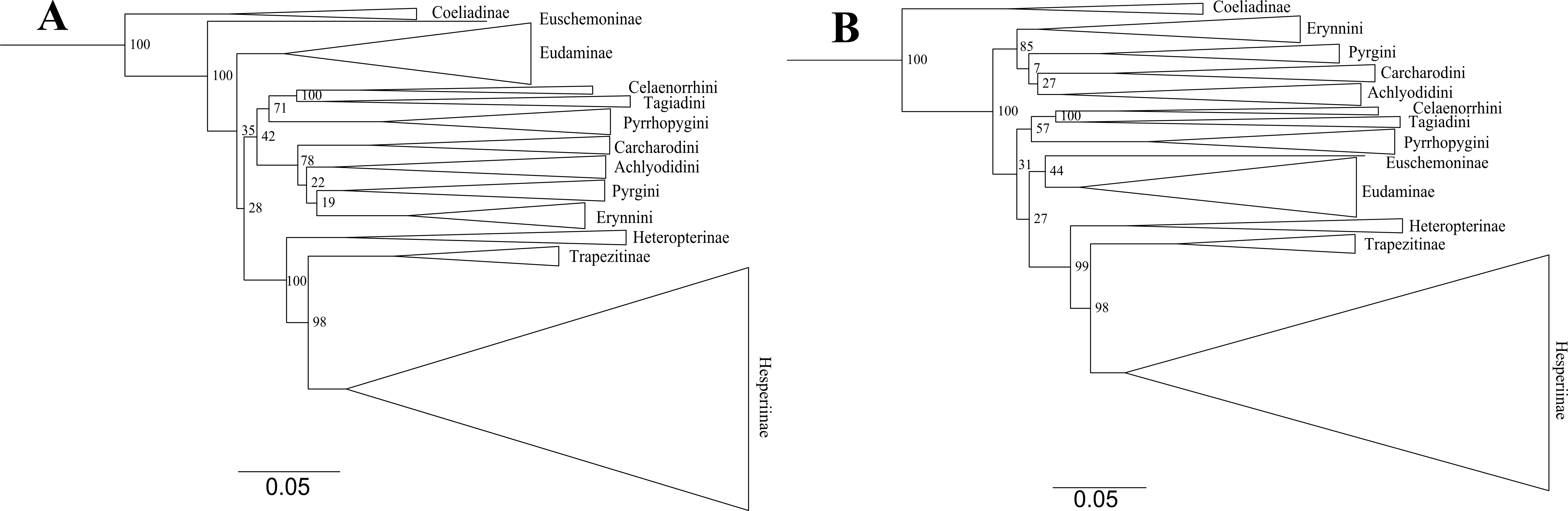

Supplement: Supplemental Information 8 — (A) Summarization of the ML-bootstrap trees from the concatenated dataset on the ML-best tree from the combined nuclear dataset. (B) Summarization of the ML-bootstrap trees from the combined nuclear dataset on the ML-best tree from the concatenated dataset. [file peerj-04-2653-s008.png]

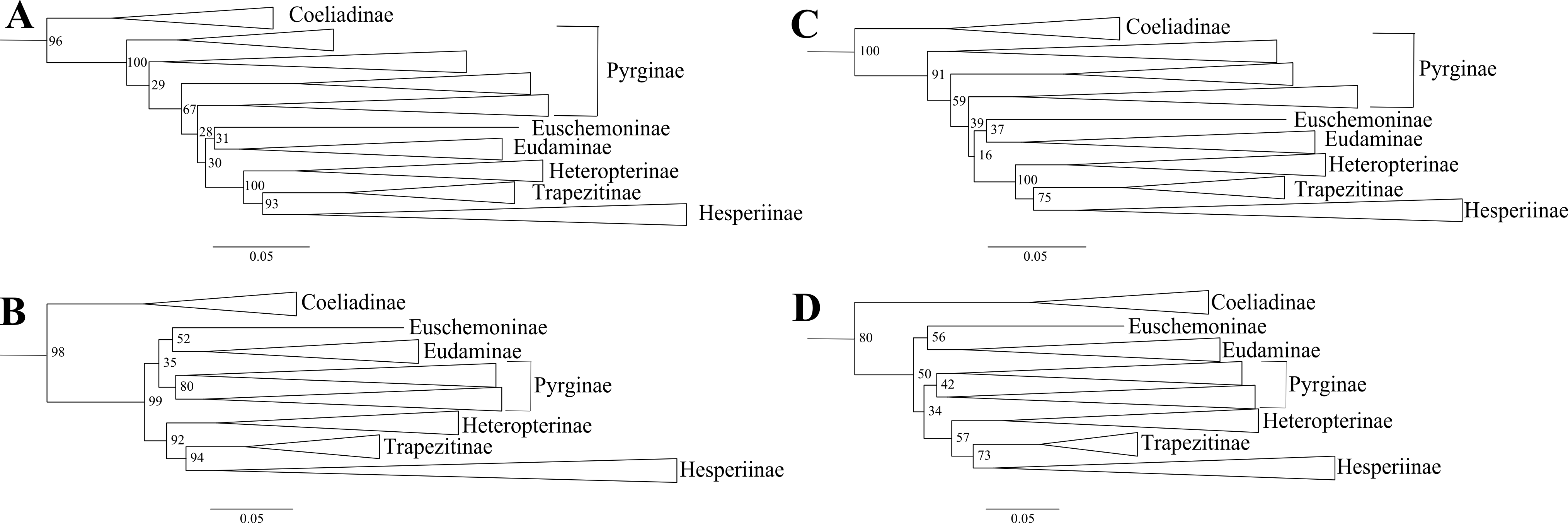

Supplement: Supplemental Information 9 — Trees from the ML analyses of different gene combinations (A) ArgKin, EF1a, GAPDH, RpS2, RpS5, wingless, (B) CAD, EF1a, IDH, wingless, (C) ArgKin, GAPDH, MDH, RpS2, RpS5 and (D) CAD, MDH using gene partitions. The node supports were derived from 1,000 bootstrap trees. The gene combinations were made based on the relationships recovered in single-gene trees for clade status of Pyrginae and sister status of Eudaminae and Euschemoninae. Thus, the expected relationships in: (A) is that Eudaminae and Euschemoninae are non-sisters, (B) Pyrginae is monophyletic, (C) Pyrginae is paraphyletic and (D) Eudaminae and Euschemoninae are sisters. [file peerj-04-2653-s009.png]

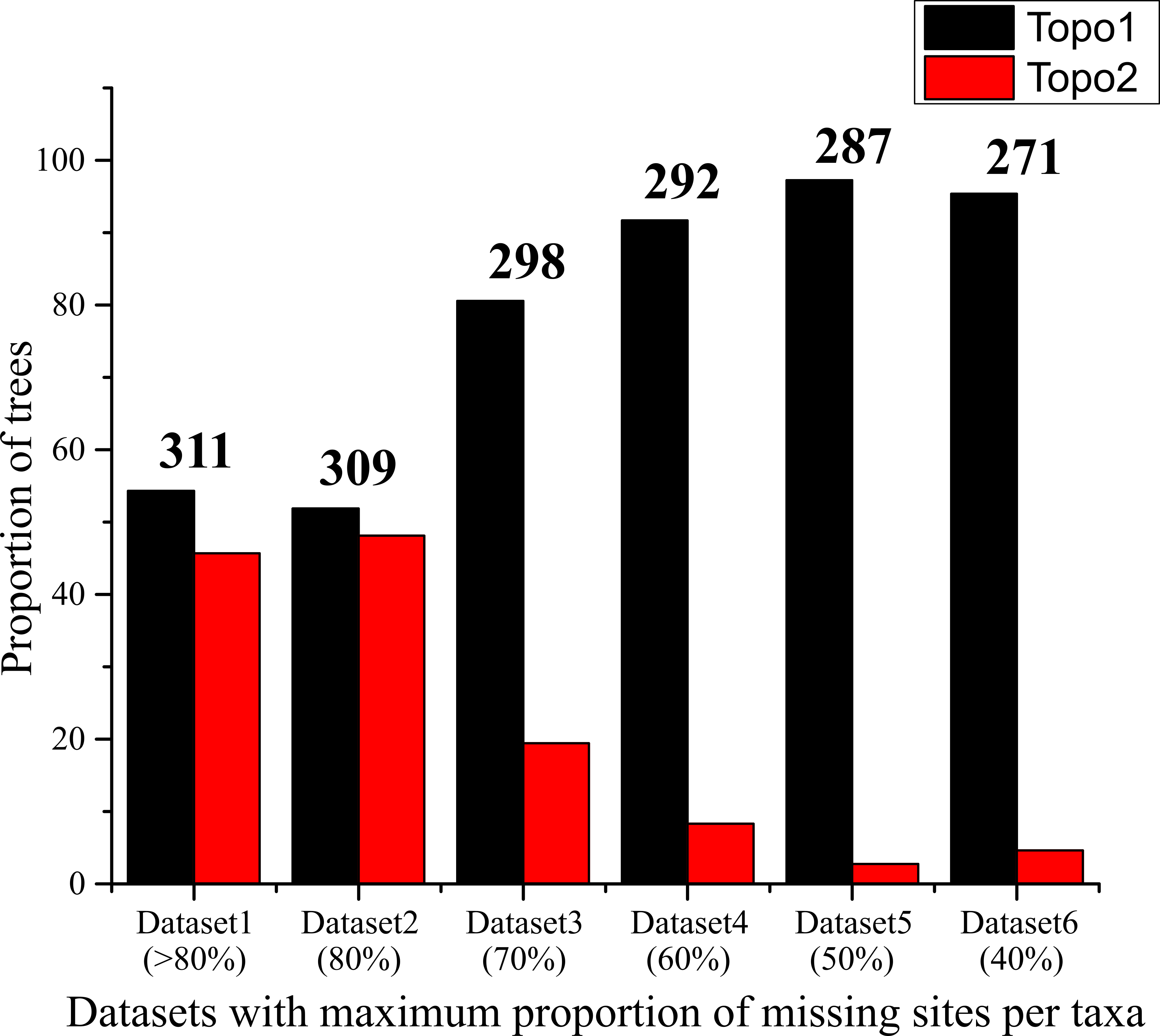

Supplement: Supplemental Information 10 — The proportion of trees, that show either topology 1 (as in Fig. 1A) or topology 2 (as in Fig. 1B), in the set of 105 or 108 independent ML trees from the analyses of different datasets as mentioned in Table S2. The number on the top of the bar represents the number of ingroup taxa in the respective dataset. [file peerj-04-2653-s010.png]

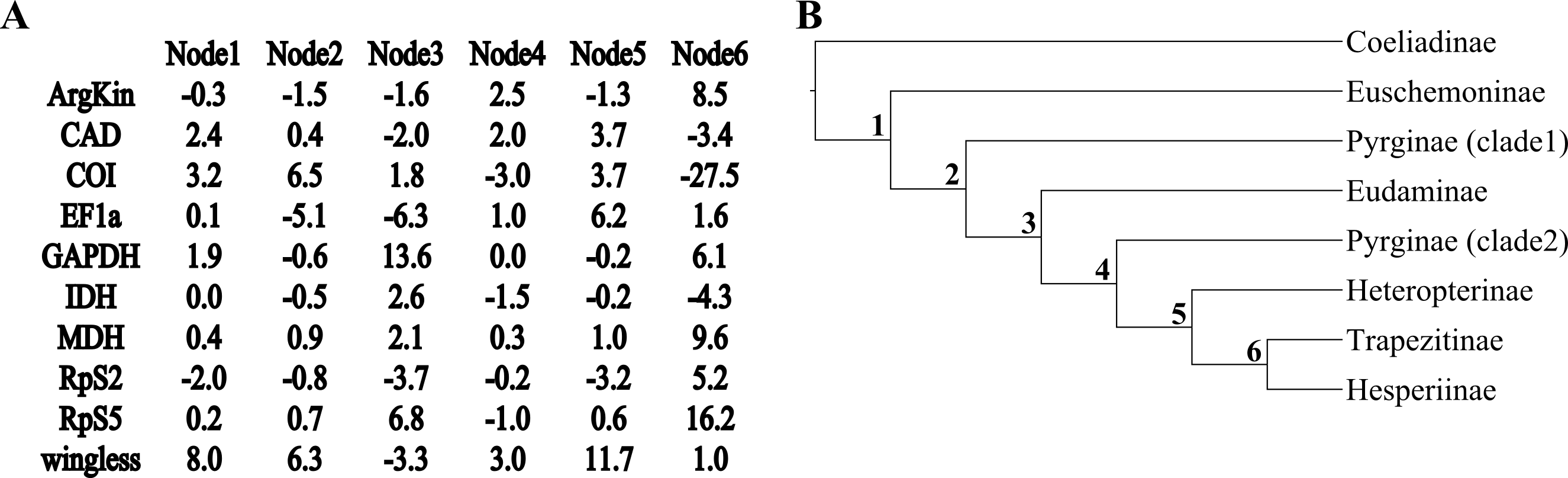

Supplement: Supplemental Information 11 — (A) The node specific partitioned bremer support for ten-gene partitions. (B) The positions of the nodes are shown on the consensus tree reconstructed using concatenated dataset. [file peerj-04-2653-s011.png]
